# Validation and reproducibility of the International Study of Asthma and Allergies in Childhood (ISAAC) Written Allergic Rhinitis Questionnaire for phone survey in children aged 6‒7 years

**DOI:** 10.1016/j.bjorl.2024.101531

**Published:** 2024-11-19

**Authors:** Priscilla Campos, Solange O.R. Valle, Antônio José Ledo Alves da Cunha, Fábio Chigres Kuschnir, Dirceu Solé

**Affiliations:** a Universidade Federal do Rio de Janeiro (UFRJ), Hospital Universitário Clementino Fraga Filho (HUCFF), Rio de Janeiro, RJ, Brazil; b Universidade Federal do Rio de Janeiro (UFRJ), Faculdade de Medicina, Rio de Janeiro, RJ, Brazil; c Universidade do Estado do Rio de Janeiro (UERJ), Departamento de Pediatria, Rio de Janeiro, RJ, Brazil; d Universidade Federal de São Paulo (UNIFESP), Departamento de Pediatria, Disciplina de Alergia Imunologia Clínica e Reumatologia, São Paulo, SP, Brazil

**Keywords:** Allergic rhinitis, Epidemiology, Validity, Reproducibility, Telephone interview

## Abstract

**Objective:**

To validate and assess the reproducibility of the ISAAC Written Allergic Rhinitis Questionnaire (WARQ) for children aged between 6 and 7 years by telephone contact.

**Methods:**

Observational study through interviews with guardians of children aged 6–7 years using the ISAAC Allergic Rhinitis (AR) module questionnaire in three different phases separated by 2 weeks each: telephone interviews in the first and third contacts and face-to-face interviews, with the same guardian of telephone interviews, in the second contact. Reproducibility was estimated using the Kappa index and validation using the sensitivity and specificity coefficients.

**Results:**

Data from 94 children (48 from the allergic rhinitis Control Group ‒ CG) were analyzed. Reproducibility showed perfect agreement (100%) for the question number 1 – Which refers to the symptoms of AR, ever: “Has your child ever had a problem with sneezing or a runny or a blocked nose when he/she did not have a cold or the flu?” and for the question number 2 – Which refers to current symptoms of AR: “In the past 12 months, has your child had a problem with sneezing or a runny or a blocked nose when he/she did not have a cold or the flu?” A strong agreement was also observed for the question number 3 (κ = 0.871) – it defines the presence of comorbidity of allergic rhinoconjuntivitis “In the past 12 months, has this nose problem been accompanied by itchy-watery eyes?” The validation showed high specificity (≥76.7%) and sensitivity (≥98%) for all questions, except for the ones related to seasonality and intensity of symptoms.

**Conclusions:**

Our results showed that the ISAAC AR module questionnaire by telephone interviews has good reproducibility and high agreement with the clinical diagnosis of AR. It may be an appropriate alternative tool in epidemiological studies of childhood AR, especially in periods of social isolation, such as Coronavirus pandemic.

**Level of evidence:**

Cohort Study. Level IV

## Highlights

• Allergic Rhinitis (AR) is the most prevalent allergic disease in Brazil.
• ISAAC stablished an epidemiological landmark in study of allergic diseases.
• ISAAC AR Questionnaire by phone has high agreement with clinical diagnosis of AR.
• ISAAC AR Questionnaire by phone discriminates children with and without AR.

## Introduction

Allergic rhinitis is the most prevalent allergic disease in Brazil, and it comprehends a symptomatic disorder of the nose triggered after allergen exposure by an Immunoglobulin E (IgE)-mediated inflammation.^1^, ^2^

There are four cardinal symptoms occurring in AR: sneezing, nasal congestion, nasal itching and anterior and/or posterior mucous discharge watery rhinorrhea. These symptoms occur during two or more consecutive days for more than one hour on most days.^1^

Despite of its great prevalence, economic impact of AR was often underestimated due to its low morbidity and mortality. However, because of AR numerous complications, high cost of chronic medical treatment, negative impact on quality of life, and association with other comorbidities such as atopic eczema and asthma, the perspective of global community about this disease has changed.^1^, ^2^, ^3^

AR is a worldwide health issue that causes major illness and disability. Brazilian data, obtained through the International Study of Asthma and Allergies in Childhood (ISAAC) study demonstrated that the prevalence of AR in children and adolescents ranges from 10% up to 7%, depending on the definition used and the age group studied.^3^

ISAAC study stablished an epidemiological landmark in the study of asthma and other allergic diseases, such as atopic eczema and AR, and allowed international and regional parallels of prevalence and risk factors associated with these conditions.^4^

In 2006, as consequence of the importance of Chronic Non-Communicable Diseases (NCDs) in the Brazilian population, the Ministry of Health implemented the Surveillance System for Risk and Protective Factors for Chronic Diseases by Telephone Survey (VIGITEL).^5^, ^6^

This system is a population-based cross-sectional survey that interviews probabilistic samples of individuals aged 18 years and older who have a telephone in their homes, using questionnaires that address risk or protective factors for NCDs. This type of surveillance system shows important advantages over traditional household surveys, such as lower cost per interview and faster data collection, with easier monitoring of the indicators studied.^5^, ^6^

In a study carried out in the city of Rio de Janeiro, Valle et al. validated the written ISAAC asthma questionnaire for children aged 6–7 years by telephone interviews, showing good agreement and reproducibility of this method when compared to the original one.^5^

Oliveira et al. also performed the same substantial results, in 2022, when compared the written ISAAC atopic eczema questionnaire to telephone interviews.^7^

The aim of this study was to validate and evaluate the reproducibility of the WARQ in children aged between 6 and 7 years administered to guardians through telephone interviews.

## Methods

This was an observational study carried out in Rio de Janeiro, Brazil, in three health units aimed at education, research, assistance, and technological development. All those institutions are tertiary hospitals that receives patients from different neighborhoods and cities.

The city of Rio de Janeiro, with 6,211,223 inhabitants (97.3% with telephone coverage), a population density of 5175.6 inhabitants/km^2^, and Human Development Index (HDI) of 0.799, is located on the southeastern region of Brazil.

This was a convenience sample. A search of medical records was carried out, and a total of 100 children aged between 6 and 7 years old, were divided into two groups. The primary criteria for inclusion in the study was to have fixed or mobile telephone lines at home. They were required to be undergoing follow-up for at least six months and be scheduled for consultation at the clinics in sequence.

The patients with AR according to the ARIA ‒ Allergic Rhinitis and Its Impact on Asthma ‒ diagnostic criteria, were also classified by its severity as ‘mild’ or ‘moderate/severe’, and by its occurrence as intermittent or persistent. This classification was established by the interviewer (the principal researcher), a specialist in Allergy and Immunology clinics, during the second phase of the study. The diagnose of AR were performed by the same researcher and confirmed with objective tests for the diagnosis of IgE-mediated allergy (skin prick test and serum-specific IgE).^1^

The “Control Group ‒ CG” consisted of children without AR or other uncontrolled allergic diseases (asthma; atopic eczema) followed at the General Pediatrics outpatient clinic, or the Dental Pediatric Department. The CG The study was carried out in 3 phases, in both groups.

**Fig. 1:**
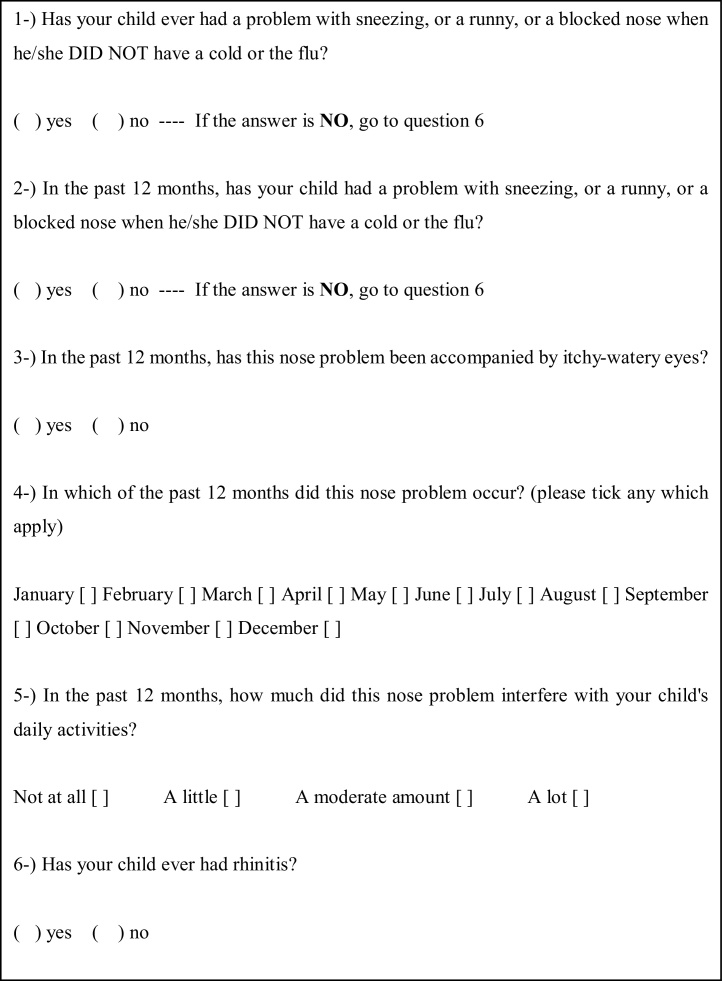
ISAAC Written Allergic Rhinitis Questionnaire (WARQ) for children between 6 and 7 years of age.

The first phase consisted in applying the WARQ (Fig. 1), previously validated for the Portuguese language by Vanna et. al., to the guardians of the children selected through telephone interviews.^9^

The main indicators assessed by this tool are the presence of AR symptoms ever and the distinction between other respiratory upper diseases as common cold, for example (“Has your child ever had a problem with sneezing, or a runny, or a blocked nose when he/she DID NOT have a cold or the flu?” – Question 1) and current symptons of AR (In the past 12 months, has your child had a problem with sneezing, or a runny, or a blocked nose when he/she DID NOT have a cold or the flu?¨ ‒ Question 2). The third question ‘‘In the past 12 months, has this nose problem been accompanied by itchy-watery eyes? – identifies the comorbidity of allergic rhinoconjuntivitis; the seasonality of the symptoms is determinate by question 4 “In which of the past 12 months did this nose problem occur?” (please tick any which apply) January [ ] February [ ] March [ ] April [ ] May [ ] June [ ] July [ ] August [ ] September [ ] October [ ] November [ ] December [ ].

Finally, we can access the severity of AR at question 5 “In the past 12 months, how much did this nose problem interfere with your child's daily activities? Not at all [ ] A little [ ] A moderate amount [ ] A lot [ ]”, and point out a prior medical diagnosis of AR at question 6 “Has your child ever had rhinitis?” Yes [ ] No [ ].^1^, ^4^, ^8^

An Informed Consent Form (ICF) were read slowly by the same interviewer (the main researcher), and after the consent of the guardian was recorded, the answers were also audiotaped directly and immediately on an electronic record.^5^, ^7^

The second phase was carried out after two weeks, coinciding with the appointment date, so that the child’s guardian could fill in the WARQ and the ICF in person under the supervision of the main researcher and a physical examination of the child were performed. This phase permitted to ascertain the absence of signs and symptoms of AR in the control group, and to maintain the routine follow-up at the AR group.

On this occasion, the children from the AR group were classified by its severity as ‘mild’ or ‘moderate/severe’, and by its occurrence as intermittent or persistent, following ARIA criteria. This face-to-face interview also allowed to rule out the presence of AR and any atopic disease, such as asthma and atopic dermatitis, by anamnesis and physical examination, in the CG group.

The third phase took place 15 days after the previous one when the same researcher conducted the second telephone interview using the WARQ. Only the data provided by the same guardians in all three stages of the study were considered for analysis of reproducibility and validation. The reproducibility of questionnaires administered by telephone was calculated using the Kappa coefficient. The results were classified as follows: above 0.81, almost perfect agreement; between 0.61 and 0.8, substantial agreement; between 0.41 and 0.6, moderate agreement; between 0.21 and 0.4, poor agreement; below 0.2, slight agreement.^9^

The validation was calculated by comparing the responses obtained during the first telephone interview with the ARIA clinical diagnostic criteria for AR used as the standard in our study.^1^, ^4^

The specificity and sensitivity for each indicator and their respective accuracy were evaluated. Afterwards, to assess the degree of agreement between the answers to the WARQ filled out by the guardians on the day of the consultation with those obtained at the first telephone interview, the Kappa coefficient was used, with the same previously mentioned classification.

The data were stored in an Excel database, and statistical analysis was performed using SPSS software, version 23. This study was approved by the Ethics Committee of the UFRJ. Written consent was replaced by verbal consent obtained prior to the interview, based on article N. 5 of Resolution N. 510, of April 7, 2016.

## Results

Between October 2020 and October 2022, 94 children were included in the study, 48 (51.1%) in the AR group and 46 (48.9%) in the Control Group (CG). Two children from the CG were excluded because they failed to respond the first phone interview and four from the AR group were excluded in the second interview due to presence of severe or uncontrolled allergic asthma or atopic dermatitis, which could behave as confounding bias for some AR symptoms. Fifty-seven patients (61%) were male, 50.9% of them were in the control group and the other 49.1% belonged to the AR group. In the AR group, mothers accounted for 89.6% of respondents and fathers and grandparents, 6.3% and 4.1% each. In the control group, mothers, fathers and grandmothers were the respondents in 80.4%, 15.2%, and 4.4% respectively. There was no statistically significant difference regarding the percentage of this proportion between the two groups.

Ascribed to the presence of more than one respondent for the same child in the different phases of the research or because the last interview was not carried out due to the new coronavirus pandemic, 7 patients (5 from the AR group and 2 from the CG) were excluded from the reproducibility assessment, and another 4 (3 from the control group and 1 from the AR group) were excluded from the agreement assessment process between the first telephone and the in-person interview. The average duration of each telephone interview was 4 minutes.

According to ARIA criteria, the AR group were classified by its occurrence in intermittent – only 10 patients (20.8%) and persistent (79.1%). Regards to severity 29 patients (60.4%) were grouped as mild AR; and 19 as moderate/severe AR (39.6%). All patients at the intermittent group were categorized as mild AR. Patients in the control group were not submitted to this analysis because they did not have signs or symptoms of AR.

The reproducibility of AR indicators of the ISAAC Written Allergic Rhinitis Questionnaire (WARQ) is showed at Table 1. The common questions responded by the control group and the AR group are the first and sixth questions. Therefore, these are the only ones considered for reproducibility at this table.

**Table 1:** Reproducibility of AR indicators of the ISAAC Written Allergic Rhinitis Questionnaire (WARQ) for children aged 6‒7 years applied through telephone interviews with their caregivers (n = 94) Rio de Janeiro, 2020‒2022.

Indicator	Agreement n (%)^a^	Kappa Coefficient (K)	95% CI	*p*-value
Symptoms ever	86 (89.4%)	0.953	0.743 – 1.167	0.000
Prior diagnosis of AR	85 (88.3%)	0.930	0.719 – 1.141	0.000

a Total number and percentage of concordant responses between the 2 telephone interviews.

The results of the validation of the WARQ obtained through the first telephone interview – in the AR group ‒ with the guardians, when compared with the clinical diagnosis given by a specialist based on the ARIA criteria for AR and confirmed with objective tests for the diagnosis of IgE-mediated allergy (skin prick test and serum-specific IgE) is showed at Table 2. The interviews showed high sensitivity (≥ 98%) and specificity (≥ 93.9%), for questions number one (“Has your child ever had a problem with sneezing”), two (“Symptoms in the last 12-months”), and six (“Has your child ever had rhinitis?”).

**Table 2:** Results of the validation of the WARQ obtained through the first telephone interview – in the AR group ‒ with the guardians, when compared with the clinical diagnosis given by a specialist based on the ARIA criteria for AR (in person interview).

Indicator	Sensitivity (%)	Specificity (%)	Accuracy (%)
Symptoms ever	98,0	100	100
Symptoms in the last 12-months	100	93.9	93.8
Itchy-watery eyes	100	76.7	70.8
Prior diagnosis of AR	100	95.8	95.8

Question number three (“nose problem been accompanied by itchy-watery eyes”) had high sensitivity (≥ 98%) and specificity (≥ 76%).

On the other hand, the fourth (“In which of the past 12 months did this nose problem occur?”) and fifth (“In the past 12 months, how much did this nose problem interfere with your child's daily activities?”) questions showed lot of divergence in the responses, which preclude us to calculate the sensitivity and specificity to these items.

When the two methods of administration (by phone and in-person) of the WARQ were compared, discriminating the agreement of responses between them, there was an almost perfect agreement (κ ranging from 0.87 to 1.00) in the first three questions (“Distinction between common cold and AR”, “Symptoms in the last 12 months”, “Itchy-watery eyes”), and substantial agreement (κ = 0.66) in the last question (Table 3).

**Table 3:** Agreement between the in-person responses to the Written Allergic Rhinitis Questionnaire (WARQ) provided by caregivers of children aged 6‒7-years and the responses obtained at the 1st telephone interview (n = 48) – AR group. Rio de Janeiro, 2020‒2022.

Indicator	Agreement n (%)^a^	Kappa Coefficient (κ)	95% CI	*p*-value
Symptoms ever	48 (100%)	Not calculated^b^	Not calculated^b^	Not calculated^b^
Symptoms in the last 12-months	48 (100%)	1.00	0.717 – 1.283	0.000
Itchy-watery eyes	45 (95.6%)	0.87	0.579 – 1.164	0.000
Prior diagnosis of AR	47 (97.9%)	0.656	0.373 – 0.939	0.000

a Total number and percentage of concordant responses between the 2 telephone interviews.

b There were not a single divergence between the responses, therefore κ could not be calculated.

## Discussion

The WARQ applied by telephone interview showed good reproducibility in our study. The reproducibility is the degree to which repeated measurements under unchanged conditions show the same results.^10^ Telephone interviews carried out with guardians of children aged between 6 and 7 years, using the WARQ demonstrated almost perfect or substantial agreement for all indicators of AR, indicating that respondents understood the questionnaire. We certified that conditions remained unchanged through applying the WARQ by the same interviewer and same responder at all phases.

The ISAAC core questions for rhinitis were incorporated into our questionnaire as they represent a widely accepted standardized tool for the assessment of the prevalence of rhinitis in children and adolescents.^4^ The WARQ is a low-cost instrument that has high sensitivity and specificity.^3^ Considering both age groups (adolescents aged between 13 and 14 years old, and children 6–7 years old) the return of filled ISAAC WQ in Brazil was in media 73%, varying from 62% to 98%.^3^

When considering adolescents only, worldwide, the ISAAC questionnaire had a return rate higher than 95%, mainly because it was filled out directly by them at school, while the return rate of the written questionnaire aimed at children between 6 and 7 years of age, which should be completed at home by their guardians, had only approximately 60% of return rate.^11^ In order to enhance data quality and reduce losses in epidemiological studies, written questionnaires can be replaced by telephone interview, which is faster, less expensive, and more practical than the traditional methods.^12^

Valle et al. validated the written ISAAC asthma questionnaire for children aged 6–7 years by telephone interviews, showing good agreement and reproducibility of this method when compared to the original one.^5^ The same was performed by Oliveira et al. regards to the written ISAAC atopic eczema questionnaire, in 2022.^7^

Questionnaire validation was estimated by comparing the acquired responses with the clinical criteria used for the diagnosis of AR, showing the limit to which, the correct answer was provided. The first and the sixth question (respectively “symptoms ever” and “prior diagnosis of AR”) have high sensitivity (proportion of subjects with AR whose answers were correct ‒ true positives) and specificity (proportion of patients without AR who provided correct answers ‒ true negatives).^10^ High sensitivity and specificity were obtained for the first three and the sixth questions, which contribute to the clinical diagnosis of recent AR. On top of that, the indicator “symptoms ever” revealed the best accuracy. The worst indicator, nevertheless, with an accuracy of 70.8%, was “Itchy-watery eyes” which identifies the comorbidity of allergic rhinoconjuntivitis.

The question “In which of the past 12 months did this nose problem occur? (please tick any which apply)” had a lot of divergence between the responders during the interviews, enabling to calculate kappa coefficient, sensitivity and specificity. The suggestion that we consider after this study is to group the months of the year into clusters; for instance: “In how many months of the year did this nose problem occurred: ( ) Less than three months ( ) Between three and six months ( ) More than six months to the whole year. Another approach would be to divide the year into seasons (spring, summer, autumn, and winter). This last propose could be, therefore, an issue in Brazil (except for the south region), because the seasons are not well defined, and there is a constant climate change in our country. These clime conditions seem to be the reason for the divergence in responses for the fifth question, in addition of a possible recall bias, which also enabled us to calculate kappa coefficient, sensitivity and specificity for this question (In the past 12 months, how much did this nose problem interfere with your child's daily activities?).

The agreement between the first telephone interview and the in-person interview was almost perfect or substantial, indicating that these two different WARQ methods had similar responses.

We had challenge limitations in this present study that can compromise the generalization of its results, such as the non-random selection, absence of sample size calculation, restriction of the age group between 6 and 7 years, which may not be representative of other age groups, and exclusive participation of patients from a tertiary hospital.

However, the clinical diagnosis of AR carried out by a specialist, confirmed with objective tests for the diagnosis of IgE-mediated allergy (skin prick test and serum-specific IgE), the presence of only one respondent at all phases of the study and the same researcher for all the phases, ensure good quality of data. We also highlight the fact that the data was collected in in similar proportions in three different tertiary hospitals, by the same researcher, which provided us a more diverse sample, from different neighborhoods of the city of Rio de Janeiro, therefore representing with more reliability this population.

## Conclusion

Our data show that the WARQ acquired through telephone interviews has good reproducibility and high agreement with the clinical diagnosis of AR made by a specialist, being effective to discriminate children with and without the disease. Consequently, it can be a proper alternative tool for epidemiological studies in AR, especially during a pandemic, such as the COVID-19, when social isolation is extremely important and in-person questionnaires become impossible to be performed.^13^

## Credit authorship contribution statement

Campos, P declares no conflict of interest; Valle SOR declares no conflict of interest; CUNHA, A. J. L. A. declares no conflict of interest; KUSCHNIR, F. C declares no conflict of interest; SOLÉ, D. declares no conflict of interest. The authors declare that they have no conflicts of interest regarding this Study.

## Funding

This research received no specific grant from any funding agency in the public, commercial, or not- for-profit sectors.

## Declaration of competing interest

The authors declare no conflicts of interest.
